# Molecular and Clinical Comparison of Enterovirus D68 Outbreaks among Hospitalized Children, Ohio, USA, 2014 and 2018

**DOI:** 10.3201/eid2511.190973

**Published:** 2019-11

**Authors:** Huanyu Wang, Alejandro Diaz, Katherine Moyer, Maria Mele-Casas, Maria Fatima Ara-Montojo, Isabel Torrus, Karen McCoy, Asuncion Mejias, Amy L. Leber

**Affiliations:** Nationwide Children’s Hospital, Columbus, Ohio, USA (H. Wang, A. Diaz, K. Moyer, M. Mele-Casas, M.F. Ara-Montojo, I. Torrus, K. McCoy, A. Mejias, A.L. Leber);; The Ohio State University, Columbus, Ohio, USA (H. Wang, A. Mejias, A.L. Leber)

**Keywords:** Enterovirus D68, EV-D68, pediatric, molecular detection, asthma, acute flaccid myelitis, children, Ohio, viruses, United States

## Abstract

Enterovirus D68 (EV-D68) causes respiratory tract infections and neurologic manifestations. We compared the clinical manifestations from 2 EV-D68 outbreaks in 2014 and 2018 and a low-activity period in 2016 among hospitalized children in central Ohio, USA, and used PCR and sequencing to enable phylogenetic comparisons. During both outbreak periods, infected children had respiratory manifestations that led to an increase in hospital admissions for asthma. The 2018 EV-D68 outbreak appeared to be milder in terms of respiratory illness, as shown by lower rates of pediatric intensive care unit admission. However, the frequency of severe neurologic manifestations was higher in 2018 than in 2014. During the same period in 2016, we noted neither an increase in EV-D68 nor a significant increase in asthma-related admissions. Phylogenetic analyses showed that EV-D68 isolates from 2018 clustered differently within clade B than did isolates from 2014 and are perhaps associated with a different EV-D68 subclade.

Enterovirus D68 (EV-D68) was originally identified in 1962 in children with severe respiratory tract infections in California, USA (*1*). The virus shares biological features with enteroviruses and rhinoviruses and was reported sporadically after these initial reports (*2*). However, EV-D68 gained epidemiologic and clinical relevance in 2014 after it was identified as an important pathogen associated with severe lower respiratory tract infections and, in some cases, with central nervous system disease (i.e., acute flaccid myelitis [AFM]) (*3*–*5*).

Nationwide Children’s Hospital (NCH) in Columbus, Ohio, USA, experienced a first outbreak of EV-D68 in 2014 that was associated with respiratory distress and disproportionately affected children with asthma; no case of AFM was identified (*3*). Although EV-D68 reportedly has a biennial seasonality, NCH did not have an EV-D68 outbreak in 2016. EV-D68 emerged again in 2018 and caused respiratory infections and, in some cases, neurologic manifestations.

The objective of this study was to compare differences in clinical characteristics and disease severity among children hospitalized with EV-D68 infection at NCH in 2018 with those identified during the 2014 outbreak and during a low-activity period (2016). We also sought to define the overlap between EV-D68 circulation and hospitalizations for asthma and compare the sequence variation of EV-D68 strains identified during the 2018 outbreak with strains identified in previous years.

## Materials and Methods

### Sample Collection and Testing Algorithms

We identified children hospitalized at NCH who had EV-D68 infection during the 2018 outbreak and during a nonoutbreak period (2016) and compared their clinical manifestations and characteristics with those identified during the 2014 outbreak as previously described (*3*). In brief, during June 1–October 19, 2018, we collected nasopharyngeal samples using flock swabs that went into viral transport media. All samples were obtained in accordance with standard of care for patients <21 years of age who tested positive for rhinovirus/enterovirus (RV/EV) on the FilmArray Respiratory Panel version 1.7 (*6*) and were stored at −80°C for further testing. We retrospectively identified samples from 2016, collected during the same period, and retrieved them from −80°C storage for testing. After excluding duplicates, we selected samples on the basis of availability, amount of remnant, and integrity for EV-D68 testing using a laboratory-developed real-time reverse transcription PCR (rRT-PCR) targeting the 5′ nontranslated region of the human enterovirus genome as described (*3*). Because we conducted EV-D68 testing after patient encounters, results were not available to the treating physician.

**Figure 1:**
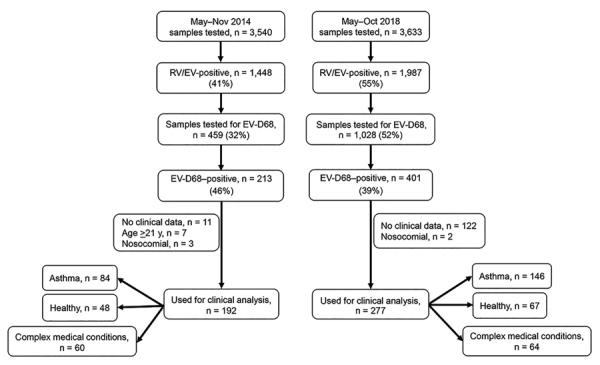
Sample and patient selection for investigation of EV-D68 outbreaks, Columbus, Ohio, USA. Viral testing was conducted at Nationwide Children’s Hospital Department of Pathology. During May–November 2014, a total of 3,540 samples underwent viral testing, of which 41% tested positive for RV/EV by a single or multiplex PCR. Four hundred fifty-nine samples were selected randomly on the basis of availability, integrity, and amount of specimen, of which 44% were positive for EV-D68. During May–October 2018, a total of 3,633 samples were tested for RV/EV by FilmArray Respiratory Panel v1.7 (*6*); 1,987 (55%) were positive. Of the 1,025 convenience samples, 401 (39%) were positive for EV-D68. After samples for which clinical data were not available, for which patient age was >21 years, or for which EV-D68 was acquired nosocomially were excluded, 192 case-patients from the 2014 outbreak and 278 from the 2018 outbreak were included in the analyses. EV-D68, enterovirus D68; RV/EV, rhinovirus/enterovirus.

The sampling selection criteria differed between the 2 outbreak periods (Figure 1). During 2014, we screened a smaller set of samples for EV-D68 and focused on hospitalized patients. In 2018, we screened samples from both outpatients and inpatients. However, for both periods the clinical analyses focused on hospitalized patients only.

We reviewed electronic healthcare records from patients positive for EV-D68 for data collection. Patients in whom EV-D68 was identified but for whom clinical data were not available, those evaluated in the outpatient setting, those >21 years of age, and patients who acquired RV/EV infection during hospitalization were excluded from analyses. We compared differences in clinical characteristics and disease severity parameters among patients from the 2018 EV-D68 outbreak, those identified in 2016, and patients during the 2014 outbreak. The clinical characteristics of patients during the 2014 outbreak were previously reported (*3*). The Institutional Review Board of NCH approved the study.

### Admissions for Asthma

We retrieved data related to admissions for asthma during the same time periods in 2014, 2016, and 2018 from the electronic data warehouse. We included patients <21 years of age who were hospitalized with an asthma diagnosis in any NCH inpatient unit. We used the following codes from the International Classification Diseases, Ninth (ICD-9) or Tenth (ICD-10) Revision, for asthma: ICD-9, 493.*; ICD-10, J45.20–J45.998.

### EV-D68 rRT-PCR Testing and Sequencing

For EV-D68 detection and quantitation, we used a laboratory-developed rRT-PCR as described previously (*3*). We selected a subset of EV-D68–positive samples for partial viral protein (VP) 1 gene sequencing of an 805-bp PCR product, as described (*7*). We performed cycle sequencing with BigDye Terminator v3.1 Cycle Sequencing Kit (Applied Biosystems, https://www.thermofisher.com) on the automated sequencer 3130xl Genetic Analyzer (Applied Biosystems) bidirectionally. We generated multiple sequence alignments and phylogenetic trees and compared the amino acid sequences (including BE, DC, and GH loops) of partial VP1 as described (*3*,*8*,*9*).

### Statistical Analysis

We conducted descriptive analyses using frequency distributions or rates and used medians and interquartile ranges to summarize the demographic data and patients’ baseline characteristics. We analyzed associations between categorical variables using the Fisher exact or χ^2^ test and assessed normality for continuous variables using the Shapiro-Wilk test and 2-tailed Student *t* tests, Mann-Whitney test, 1-way ANOVA (analysis of variance), or Kruskal-Wallis tests where used as appropriate. Two-tailed p values <0.05 were considered significant. We performed statistical analyses using GraphPad Prism 8 (https://www.graphpad.com).

## Results

### Sample Selection and Clinical Characteristics of EV-D68–Infected Patients during the 2018 Outbreak

During the 2018 outbreak, of 3,633 samples tested by the FilmArray panel 1,987 (55%) were positive for RV/EV. We further evaluated 1,028 samples, of which 401 (39%) tested positive for EV-D68. This number compares with 213 (46%) EV-D68–positive samples of 459 in the 2014 outbreak (Figure 1).

Of the 401 patient samples that tested positive for EV-D68 in 2018, we excluded 124 (122 because patients were evaluated in the outpatient setting or clinical data were not available and 2 from children with nosocomial EV-D68 infection), leaving a total of 277 patients hospitalized with EV-D68 infection. Of those, 67 (24%) children were previously healthy, 146 (53%) had preexisting asthma or a history of wheezing, and 64 (23%) had another underlying chronic medical condition (Table 1). Children with a history of asthma or wheezing were older (median age 4.1 years) than children who had complex medical conditions (median 2.5 years) or were previously healthy (median 1.4 years; p<0.01); findings did not differ by sex or race. Most children had respiratory symptoms (94%–100%) with or without fever, followed by gastrointestinal manifestations (27%–32%). Eight (2.9%) children had neurologic manifestations, 2 AFM, and 1 opsoclonus/myoclonus syndrome (OMS). 

**Table 1:** Demographic and clinical characteristics of children with EV-D68 infection, Nationwide Children’s Hospital, Columbus, Ohio, USA, 2018*

Characteristics	Previously healthy, n = 67	Chronic medical condition, n = 64	Asthma, n = 146	p value
Median age, y (IQR)	1.4 (0.9–4.0)	2.5 (1.1–6.9)	4.1 (2.1–7.5)	**<0.0001**
Sex, no. (%)				
M	31 (46.3)	36 (56.2)	84 (57.5)	0.29
F	36 (53.7)	28 (43.8)	62 (42.5)	
Race, no. (%)				
White	42 (62.7)	43 (67.2)	84 (57.5)	0.12
Black	14 (20.9)	13 (20.3)	50 (33.5)	
Other	11 (16.4)	8 (12.5)	13 (8.9)	
PICU admission, no. (%)	25 (37.3)	19 (29.7)	92 (63)	**<0.0001**
Median hospitalization, d (IQR)	1.8 (1–3)	3.2 (1.5–8)	2.5 (1.7–3.5)	**0.003**
Signs/symptoms, no. (%)				
Respiratory	64 (95.5)	60 (93.8)	146 (100)	**0.01**
Fever	39 (58.2)	36 (56.3)	67 (45.8)	0.16
Neurologic	4 (5.9)	3 (4.7)	1 (0.7)	0.06
Gastrointestinal	18 (26.8)	20 (31.3)	40 (27.4)	0.81
Rash	3 (4.5)	0	1 (0.7)	0.05
Median EV-D68 C_t_ (IQR)	26.1 (22.2–30.3)	25.5 (22.2–28.8)	25.1 (22–38.5)	0.67

*Bold indicates significance. C_t_, cycle threshold; EV-D68, enterovirus D68; IQR, interquartile range; PICU, pediatric intensive care unit.

Overall, children with asthma required pediatric intensive care unit (PICU) admission more frequently (63%) than did previously healthy children (37%) or children with chronic medical conditions (29%; p<0.0001); however, duration of hospitalization was longer for children with underlying conditions. The EV-D68 semiquantitative viral load (cycle threshold) ranged from an average of 25.1 to 26.1 and did not differ significantly between groups.

### Clinical Manifestations during the 2014 and 2018 EV-D68 Outbreaks and the 2016 Low-Activity Period

We compared the demographic and clinical characteristics of children from the 2014 and 2018 EV-D68 outbreaks (Table 2). Overall, children with EV-D68 infection identified in 2018 were significantly younger and more often of white race; we found no differences in sex or presence of asthma or other chronic medical conditions. During the 2018 outbreak, children with EV-D68 infection more commonly had gastrointestinal symptoms than during the 2014 outbreak (28% in 2018 vs. 12% in 2014; p<0.001); symptoms included emesis, abdominal pain, and diarrhea. On the other hand, children with EV-D68 infection identified during the 2014 outbreak had respiratory symptoms and skin rashes more frequently than did children during the 2018 outbreak. The proportion of children who required PICU admission was lower in 2018 (49%) than in 2014 (68%; p<0.0001), and duration of hospitalization was shorter in 2018 (2.5 days) than in 2014 (2.8 days; p = 0.01).

**Table 2:** Demographic and clinical characteristics of children with EV-D68 infection, Nationwide Children’s Hospital, Columbus, Ohio, USA, 2014 and 2018 outbreaks*

Characteristic	2014, n = 192	2018, n = 277	p value
Demographics			
Median age, y (IQR)	5 (2–7.7)	3.1 (1.2–6.8)	0.0008
Sex, no. (%)			
M	112 (58.3)	153 (55.2)	0.50
F	80 41.7)	124 (44.6)	
Race, no. (%)			
White	79 (41.1)	169 (61.0)	
Black	75 (39.1)	76 (27.4)	
Other	38 (19.8)	32 (11.5)	**<0.0001**
Illness, no. (%)			
Previously healthy	48 (25)	67 (24.2)	0.09
Chronic condition†	60 (31.2)	64 (23.1)	
Asthma	84 (43.8)	146 (52.7)	

Clinical presentation and disease severity, no. (%)			
Respiratory	192 (100)	271 (97.8)	0.08
Fever	106 (55.2)	142 (51.3)	0.45
Neurologic	4 (2.1)	8 (2.9)	0.57
AFM/OMS	0	3 (1.1)	0.27
Other: seizures	4 (2.1)	5 (1.8)	>0.99
Gastrointestinal	24 (12.5)	79 (28.5)	**<0.0001**
Rash	10 (5.2)	4 (1.4)	**0.02**
PICU admission, no. (%)	131 (68.2)	136 (49.1)	**<0.0001**
Hospitalization, d (IQR)	2.8 (1.9–4.2)	2.5 (1.6–3.8)	**0.01**

*Bold indicates significance. AFM/OMS, acute flaccid myelitis/opsoclonus/myoclonus syndrome; EV-D68, enterovirus D68; IQR, interquartile range; PICU, pediatric intensive care unit. †Comprises genetic syndromes, congenital heart disease, history of prematurity, neurologic disorders, neuromuscular disorders.

Severe neurologic manifestations occurred more often during the 2018 outbreak. In the 2014 cohort, 4 (2%) patients had febrile seizures, but no other neurologic findings were documented, and no case of AFM was identified. In 2018, however, 8 (2.9%) patients had neurologic manifestations; 2 had AFM and 1 OMS. Two of these 3 children were previously healthy; 1 had underlying asthma. Four additional children sought treatment for complex febrile seizures (3 of these patients had a history of epilepsy), and 1 infant had viral meningitis. In this infant, parechovirus was identified by rRT-PCR in cerebrospinal fluid (Appendix Table 1).

During 2016, of 3,098 samples tested by the FilmArray panel, 1,293 (42%) were positive for RV/EV. Of those, 211 were further tested for EV-D68, and 14 (7%) yielded positive results. Nine of the 14 patients identified with EV-D68 infection were hospitalized; all had respiratory symptoms, and none had neurologic manifestations. Equal proportions of children had asthma or chronic medical conditions or were previously healthy (3 [33%] each).

### EV-D68 Seasonality and Asthma

**Figure 2:**
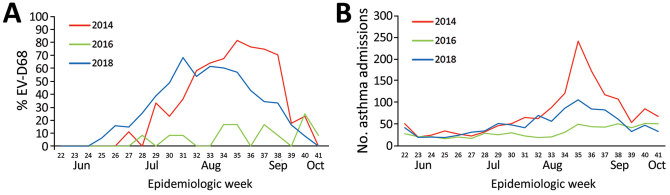
Percentage of EV-D68 (A) and number of admissions for asthma per 1,000 hospital admissions (B) among rhinovirus/enterovirus-positive (RV/EV) samples, Nationwide Children’s Hospital, Columbus, Ohio, USA, June–October 2014, 2016, and 2018. EV-D68, enterovirus D68.

We also compared the proportion of samples in which we detected EV-D68 during June–October 2014, 2016, and 2018 and analyzed the seasonality of EV-D68 in relation to admissions for asthma (Figure 2). The proportion of RV/EV detected from all nasopharyngeal samples analyzed according to the standard of care was 41% in 2014 and 2016 and 55% in 2018; overall EV-D68 detection was 44% in 2014, 7% in 2016, and 39% in 2018. The proportion of EV-D68 detected among RV/EV-positive samples (Figure 2, panel A) and the number of admissions for asthma (Figure 2, panel B) were calculated weekly during the 3 periods. The duration of the 2014 outbreak was shorter (mid-July through early October, 13 weeks), and peaked the last week of August, coinciding with a disproportionate number of admissions for asthma. In 2018, the first cases of EV-D68 were identified earlier (mid-June) and ended the first week of October (total of 16 weeks), peaking the last week of July/first week of August. In parallel, the number of admissions for asthma increased during July and August but peaked during the last week of August, which was delayed in relation to EV-D68 circulation. Admissions for asthma during the same time period in 2016 were substantially lower, as was EV-D68 detection.

### Molecular Characteristics of EV-D68 2018 Strains

We sequenced 130 EV-D68–positive samples from 2018 and aligned them to both NCH strains from prior years and to EV-D68 sequences available at the National Center for Biotechnology Information website. The NCH 2018 strains were >98.5% identical to each other and demonstrated >85% sequence identity to the VP1 regions of the prototype Fermon strain (GenBank accession no. NC_038308). These 2018 strains were also 92%–94% identical to the 2014 and 2011 NCH strains previously reported (*3*). The most closely related sequences to the NCH 2018 strains were those isolated in 2016 and 2015 from different geographic regions (*10*,*11*).

**Figure 3:**
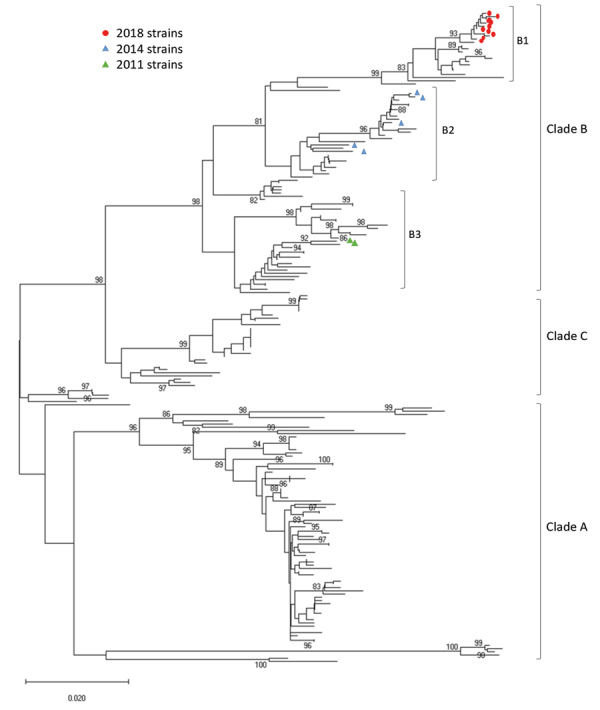
Phylogenetic analysis of EV-D68 from samples from children at Nationwide Children’s Hospital, Columbus, Ohio, USA, 2011, 2014, and 2018. Phylogenetic tree was constructed using partial viral protein 1 gene sequences. Scale bar indicates changes in base substitutions per site. EV-D68, enterovirus D68.

**Figure 4:**
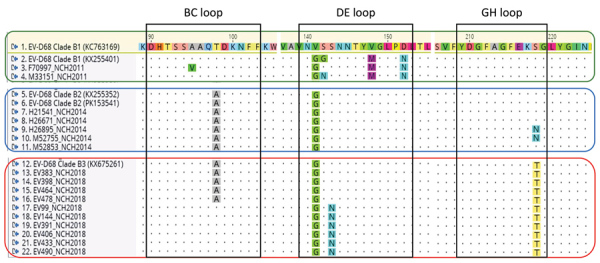
Amino acid analysis of 17 EV-D68 strains from samples from patients at NCH, Columbus, Ohio, USA, 2011, 2014, and 2018. EV-D68 strains represent subclades B1, B2, and B3 were aligned. Black boxes indicate amino acids included in viral protein 1 motifs corresponding to protein loops; colored boxes indicate strains corresponding to each subclade (green, clade B1; blue, clade B2; red, clade B3). GenBank accession numbers are given in parentheses. EV-D68, enterovirus D68; NCH, Nationwide Children’s Hospital.

We used 17 NCH strains (10 from 2018, 5 from 2014, and 2 from 2011) for further genetic characterization. Phylogenetic analyses followed by bootstrap analyses indicated that all NCH strains identified during the 2018 outbreak clustered into a new sublineage within major clade B, differently from the 2011 and 2014 NCH strains (Figure 3). Amino acid sequence alignment for the BC, DE, and GH loops (Figure 4) showed that the NCH 2018 strains displayed a unique amino acid signature, and all contained the amino acid residue (218T) that characterizes the EV-D68 clade B3 (*10*).

## Discussion

The recent emergence of EV-D68 as a cause of severe respiratory disease, coupled with its association with AFM, suggests that a deeper understanding of this virus is needed (*12*–*14*). In this study, we examined >1,000 patient specimens from 2 outbreaks and from 1 period with low EV-D68 activity and conducted both molecular and clinical analyses to compare these periods. Although the 2018 EV-D68 outbreak appeared to be milder, as shown by the lower number of hospital admissions for asthma and lower rates of PICU admissions, we observed severe neurologic manifestations only in 2018.

The comparative clinical analyses between outbreaks showed that hospitalized children during 2018 were younger, but the proportion with underling medical conditions, including asthma, was comparable between periods. Symptoms also were similar in the 2 outbreaks, with the notable exceptions of greater gastrointestinal manifestations in 2018, as well as 3 children with severe neurologic manifestations (2 with AFM and 1 with OMS), which we did not observe during 2014 or 2016.

Since 2014, concurrent with the surge of EV-D68 respiratory-associated illness, children with severe neurologic manifestations have been reported in the United States and elsewhere; episodic increases were identified in 2016 and 2018 (*5*,*12*,*14*–*21*). The cause of AFM has not been established in most cases, despite extensive pathogen-specific or metagenomic sequencing tests. Although a direct link between AFM and EV-D68 has not been established, observational and animal studies suggest a strong association. On the one hand, EV-D68 causes paralytic myelitis in mice; AFM cases have been shown to cluster during periods of EV-D68 circulation, and EV-D68 has been the most common virus detected in respiratory specimens from children with AFM, albeit rarely in cerebrospinal fluid (*22*–*25*). Other neurologic conditions associated with EV-D68 have been described, but to our knowledge, no other cases of OMS have been reported in the literature (*26*,*27*). Further studies are ongoing, but our findings agree with others and highlight the importance of comprehensive surveillance and research to further characterize the role of EV-D68 in AFM that will enable pursuit of effective therapies and prevention strategies.

Although the EV-D68 rRT-PCR testing in this study was not designed to determine true incidence, it did help to monitor EV-D68 activity and showed a marked increase in EV-D68 circulation during the summers of 2014 and 2018 and little or no activity during the same period in 2016. The increase in EV-D68 activity during 2014 and 2018 mirrored an increase in the number of admissions for asthma during those periods, and although the 2014 outbreak had a sharper increase at the end of August, the duration of the 2018 outbreak was longer. Nonetheless, in both periods admissions for asthma were significantly higher than during 2016. Although reported to have a biennial seasonality (*20*,*24*,*28*–*31*), the peaks of EV-D68 in 2014 and 2018 were related to an increase in summertime hospitalizations for asthma, which agrees with a recent study conducted in Japan (*32*). During the same period in 2016, admissions for asthma were low and no peak was observed, nor was there evidence of high EV-D68 circulation based on surveillance testing in our laboratory, which differs from data reported from other states (*28*,*33*). The magnitude of the increase in asthma hospitalizations was higher in 2014 than in 2018 (peak of 128 admissions during the peak week in 2014 vs. 61 during 2018). Whether these decreases in EV-D68 activity and severity are continued in subsequent EV-D68 outbreaks in the population studied here needs be determined. Nevertheless, EV-D68 should be suspected when summertime admissions related to asthma increase above baseline.

Phylogenetic analyses showed that EV-D68 isolates from 2014 and 2018 clustered differently within clade B (*10*,*29*,*34*,*35*). The relationship of these changes in sequence and the pathogenicity of the virus are unclear (*36*). Nevertheless, at NCH, no AFM cases were identified in 2014, but 2 AFM cases and 1 OMS case occurred in 2018. The pathogenicity and virulence of this new clade needs to be monitored and confirmed by active surveillance, which was implemented at NCH after the 2014 outbreak. Based on our experience, we have made the EV-D68 rRT-PCR test available for respiratory specimens in real time. This test will be used on the basis of clinician orders particularly as it relates to unexplained acute paralysis/muscle weakness.

This study has limitations. We did not test every specimen that was RV/EV-positive for EV-D68; we did, however, test >40% of all samples that tested positive for RV/EV during these 2 outbreaks and a low-activity period, which provides a good representation of EV-D68 circulation during those periods. The clinical analysis was limited to inpatients, thus possibly biasing the apparent severity of the EV-D68 infections; however, data were comparable between the 3 periods, which was the main study objective. Unfortunately, the samples associated with the 2 AFM cases were not available for sequencing, and thus we cannot make any definitive conclusions about different or more pathogenic viral strains.

In summary, EV-D68 circulation was associated with a significant medical burden. By more consistent and specific testing for EV-D68, a better understanding of the epidemiology of this emerging virus will help inform clinical care (*37*).

Appendix Enterovirus D68–positive patients who had neurologic manifestations during the 2018 outbreak, Nationwide Children’s Hospital, Columbus, Ohio, USA.
