# Supplementary material for: Molecular and Clinical Comparison of Enterovirus D68 Outbreaks among Hospitalized Children, Ohio, USA, 2014 and 2018
Source: Emerg Infect Dis. 2019 Nov;25(11):2055–63. doi: 10.3201/eid2511.190973 (PMC6810223; doi:10.3201/eid2511.190973)
Supplement: Appendix — Enterovirus D68–positive patients who had neurologic manifestations during the 2018 outbreak, Nationwide Children’s Hospital, Columbus, Ohio, USA. [file 19-0973-Techapp-s1.pdf]

Article DOI: https://doi.org/10.3201/eid2511.190973

Molecular and Clinical Comparison of Enterovirus D68 Outbreaks among Hospitalized Children, Ohio, USA, 2014 and 2018

Appendix

Appendix Table. Enterovirus D68–positive patients who had neurologic manifestations during the 2018 outbreak, Nationwide Children's Hospital, Columbus, Ohio.

Case	Age, y/sex	Race	Past medical history	Clinical presentation	Discharge diagnosis	Viral detection	Bacteria	Antiviral or immunomodulation Therapy	Imaging	PICU admission	Duration hospitalization, d	Outcome
1	0.09/F	Black	Perinatal HIV exposure (receiving AZT)	Fever & irritability	Viral meningitis	EV-D68 (NP) + Parechovirus (blood, CSF, superficial)	No	No	No	No	2.75	Alive
2	2.5/F	Black	No	Fever, URI‡ symptoms and seizures	Complex febrile seizure with URI‡	EV-D68 (NP)	No	No	No	No	0.92	Alive
3	6.7/F	White	Nager syndrome	Fever, URI‡ symptoms, increased seizure activity	Seizures URI‡	EV-D68 (NP)	No	No	No	Yes	3.74	Alive
4	1.8/M	White	Chromosome 2q24 deletion syndrome, Dravet syndrome	Fever, increased seizure activity, respiratory distress	Seizures Pneumonia	EV-D68 (NP)	No	No	No	No	1.24	Alive
5	0.6/M	Black	Gomez-Lopez-Hernandez syndrome	Fever, seizures, URI‡ symptoms	Seizures URI‡	EV-D68 (NP)	No	No	No	No	1.82	Alive
6	4.0/F	White	No	Fever, GI symptoms, difficulty walking	Opsoclonus -mioclonus syndrome	EV-D68 (np†)	No	Methilprednisone for 5 d	Brain MRI normal	No	6.60	Alive

Case	Age, y/sex	Race	Past medical history	Clinical presentation	Discharge diagnosis	Viral detection	Bacteria	Antiviral or immunomodulation Therapy	Imaging	PICU admission	Duration hospitalization, d	Outcome
7	8.4/M	Other	No	Fever, upper limb weakness, headache	Viral myelitis	EV-D68 (NP)	ETT culture MSSA	IVIG 2gr/kg #2	Spine MRI: diffuse spine central gray abnormality	Yes	84	Alive
8	11.8/M	White	Asthma	Fever, arm weakness, URI‡ symptoms	Viral myelitis	EV-D68 (NP)	No	IVIG 1gr/kg #1	Spine MRI: Extensive gray matter abnormality	No	4.24	Alive

*AZT, zidovudine; CSF, cerebrospinal fluid; ETT, endotracheal tube; EV-D68, enterovirus D68; GAS, group A *Streptococcus*; GI, gastrointestinal; IVIG, intravenous immunoglobulin; MRI, magnetic resonance imaging; MSSA, methicillin-susceptible *Staphylococcus aureus*; NP, nasopharyngeal swab sample; PICU, pediatric intensive care unit; URI, upper respiratory tract infection; +, positive.
